# A Case of Heyde’s Syndrome With Subvalvular Aortic Stenosis

**DOI:** 10.7759/cureus.31723

**Published:** 2022-11-21

**Authors:** Brandon L Walker, Melina C Williman, Mayank Patel, Mateo C Houle, Jason M Thomas

**Affiliations:** 1 Pulmonary and Critical Care, San Antonio Uniformed Services Health Education Consortium, San Antonio, USA; 2 School of Medicine, Uniformed Services University of the Health Sciences, Bethesda, USA; 3 Cardiology, San Antonio Uniformed Services Health Education Consortium, San Antonio, USA

**Keywords:** left ventricular outflow obstruction, echocardiogram, cardiac magnetic resonance, mechanical shear forces, gastrointestinal arteriovenous malformations, acquired von willebrand disease, subvalvular aortic membrane, subvalvular aortic stenosis, heyde’s syndrome

## Abstract

Heyde’s syndrome is a constellation of severe aortic stenosis, gastrointestinal arteriovenous malformations (AVMs), and an acquired von Willebrand type 2A coagulopathy resulting in moderate-to-severe gastrointestinal bleeding. Additional cardiac lesions have been observed to cause Heyde’s syndrome including aortic regurgitation, mitral regurgitation, aortic/mitral valve prosthetic dysfunction, ventricular septal defects, hypertrophic cardiomyopathy, left ventricular assist devices, and extracorporeal life support devices. Repairing the cardiac lesion or removing the device decreases the incidence of gastrointestinal bleeding by normalizing the acquired von Willebrand coagulopathy and decreasing the amount of gastrointestinal AVMs likely to bleed.

We describe a case of a 67-year-old woman found to have Heyde’s syndrome arising from a subvalvular aortic membrane resulting in severe subaortic stenosis with no other significant cardiac lesion. She underwent successful resection of the membrane with septal myectomy, relieving the severe subaortic stenosis and resolving her anemia. Years later, she re-presented with severe gastrointestinal bleeding from gastrointestinal malformations. Early recognition of these cardiac lesions with gastrointestinal bleeds may help decrease the morbidity and mortality that Heyde’s syndrome portends and provide evidence for early intervention.

## Introduction

In 1958, Dr. Edward C. Heyde first described an association between calcific aortic stenosis and gastrointestinal bleeds without a clear unifying mechanism in a letter to the editor in the New England Journal of Medicine [1]. In the 1970s and 1980s, associations were made between Heyde’s findings, gastrointestinal angiodysplasia, and decreased high molecular weight multimers. Reports where interventions were aimed specifically at treating the gastrointestinal bleeding had bleeding recurrences. However, when the aortic valve was replaced, bleeding was alleviated in the majority of cases. Interestingly, replacing the aortic valve alleviated the bleeding in >90% of cases. In the early 2000s, the syndrome was further described to include severe aortic stenosis inducing an acquired von Willebrand coagulopathy due to shear stress and gastrointestinal arteriovenous malformations (AVMs) causing moderate to significant bleeding [2]. An estimated 7-24% of patients with unknown origin of gastrointestinal bleeding were later found to have aortic stenosis [3]. Of patients found to have aortic stenosis with gastrointestinal bleeding, acquired von Willebrand syndrome is seen in up to 67% [4].

Severe aortic stenosis is defined as an aortic valve area ≤1.0 cm2, with an aortic velocity ≥4.0 m/s, and/or a mean transvalvular gradient ≥40 mmHg. Generally a disease of the elderly, the prevalence of aortic stenosis varies from 0.2% at ages 50-59 years, to 1.3% at ages 60-69, 2.9% at ages 70-79, and 9.8% at ages 80-89 [5]. The classic clinical manifestations are heart failure, syncope, and angina. These manifestations are described in the context of cardiac symptoms. 

von Willebrand factor (VWF) is involved in hemostasis and thrombosis. The factor is produced and processed within endothelial cells and megakaryocytes, then secreted into the plasma as long multimers. These string-like multimers attach to the endothelium where they await further cleavage. A change in configuration in the multimers by high shear stress, like those created by stenotic valves, triggers an interaction with a disintegrin and metalloprotease with a thrombospondin type 1 motif, member 13 (ADAMTS 13), causing subsequent cleavage of multimers to a smaller size [6]. Upregulated activity by ADAMTS 13 with VWF multimers occurs with situations that increase shear stress forces on VWF multimers. This further decreases their size and increases the likelihood for bleeding [6]. Cardiac lesions and/or devices as previously described create a pro-shearing force state on the involved VWF multimers causing a coagulopathy [6,7]. Furthermore, VWF multimers have an inhibitory function in angiogenesis. Disruption in VWF homeostasis may promote proliferation of AVMs. 

Angiodysplasia is a buildup of dilated vessels in the subepithelial tissue that becomes susceptible to easy bleeding. These dilated vessels tend to develop through the gastrointestinal tract in Heyde’s syndrome from the stomach to the rectum [8]. Angiogenesis begins with endothelial migration and proliferation. There is subsequent maturation with surrounding pericytes and/or vascular smooth muscle cells. Growth factors play a major role in the development of this process which include vascular endothelial growth factor (VEGF), angiopoietin-1, and angiopoietin-2. VEGF promotes endothelial proliferation and migration. Angiopoietin-1 is involved with maturation through activation of its receptor, Tie-2. The function of angiopoietin-1 is antagonized by angiopoietin-2. In states of ischemia, VEGF and angiopoietin-2 are generated. Thus, proliferation and migration with poor maturation creating AVMs is believed to occur [7-10]. Between this physiology and the reduced inhibition of VWF due to an acquired deficiency, patients with severe aortic stenosis are at high risk for developing angiodysplasia [8].

Here, we discuss a case of subvalvular severe aortic stenosis due to a subvalvular membrane with gastrointestinal AVMs and severe gastrointestinal bleeding. This case was previously presented as a poster at the Mayo Clinic 4th Annual Multimodality Cardiac Imaging Conference in November 2018.

## Case presentation

**Figure 1:**
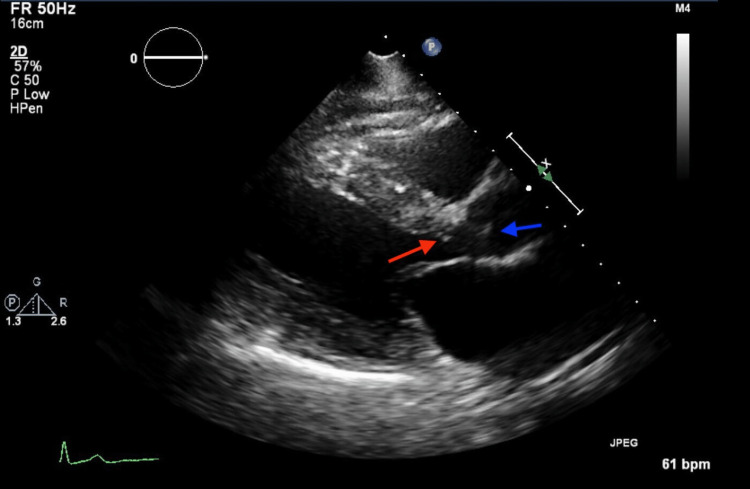
Transthoracic echocardiogram (parasternal long axis view) showing subaortic membrane (red arrow) and aortic valve leaflets (blue arrow).

**Figure 2:**
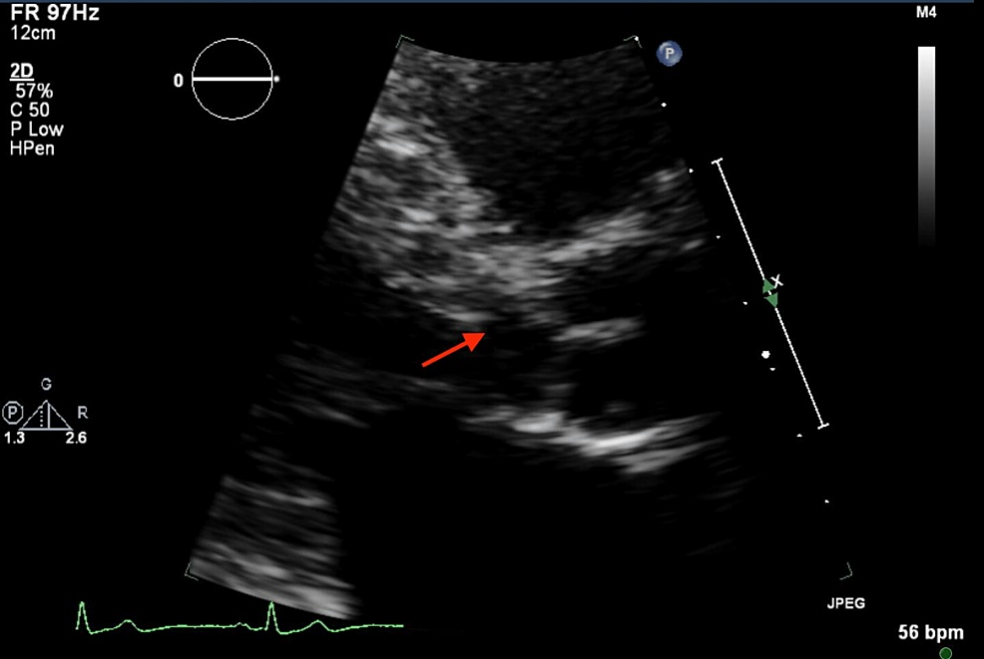
Transthoracic echocardiogram (parasternal long axis view, zoomed in) showing subaortic membrane (red arrow).

**Figure 3:**
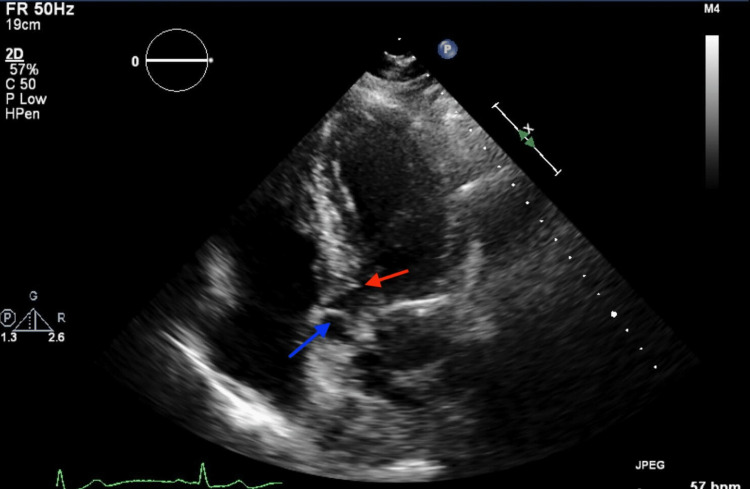
Transthoracic echocardiogram (apical 5 chamber view) showing subaortic membrane (red arrow). Aortic valve leaflets are indicated by the blue arrow.

**Figure 4:**
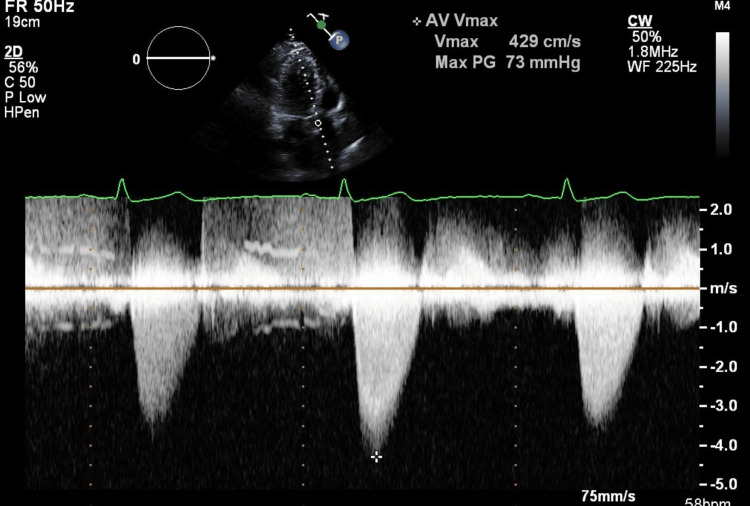
Transthoracic echocardiogram (apical 3 chamber view) with Doppler measurements across the aortic valve, demonstrating peak velocity of 4.29 m/s.

**Figure 5:**
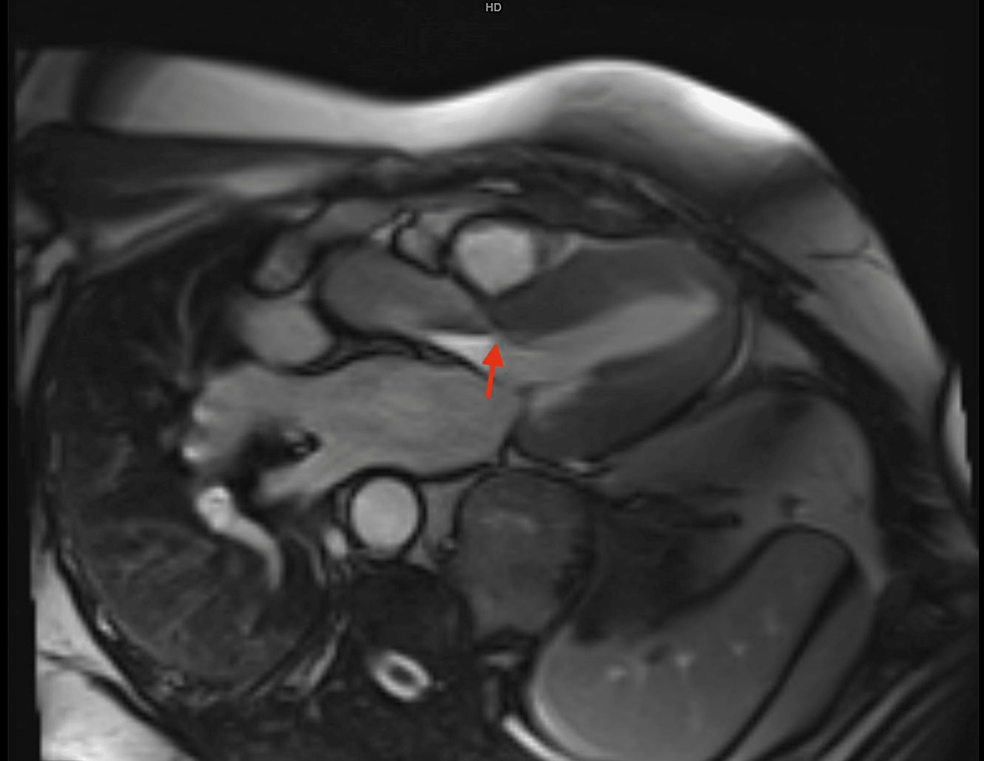
Cardiac MRI axial slice showing subaortic membrane (red arrow).

This case involves a 67-year-old woman who initially presented with worsening fatigue and dyspnea for six-to-eight weeks duration. She had no significant cardiac family history with no history of sudden cardiac death. Physical examination was remarkable for a 3/6 crescendo systolic murmur heard best at the right upper sternal border. Transthoracic echocardiogram revealed a subvalvular aortic membrane (Figures 1, 2, 3), peak velocity of 4.29 m/s across the membrane and peak gradient 73 mmHg (Figure 4). The patient’s native aortic valve area was 1.5 cm^2^. A transesophageal echocardiogram as well as cardiac MRI (Figure 5) confirmed an interventricular septal membrane 1.4 cm in length with 0.6 cm extending into the left ventricular outflow tract. The patient concomitantly was observed to have gastrointestinal bleeds due to endoscopically identified AVMs, though did not receive any endoscopic intervention. Her hemoglobin level dropped to a nadir of 4.5 g/dL and she required numerous packed red blood cell transfusions. A resection of the subaortic membrane and a septal myectomy were successfully performed without complication with a noted decrease in the gradient to 15 mmHg intraoperatively and subsequent resolution of her anemia.

There were no reported symptoms until three years later with recurrence of gastrointestinal bleeding. Her hemoglobin level dropped to 4.7 g/dl. Transthoracic echocardiogram revealed no subvalvular aortic membrane, peak velocity of 3.8 m/s across the valve, mean gradient 30 mmHg, aortic valve area of 1.1, and mild to moderate aortic valve regurgitation. von Willebrand assays were obtained which displayed a VWF ristocetin cofactor level of 98 (normal 50-150) and VWF antigen level of 157 (normal 50-150) with a ratio of 0.62 (abnormal <0.7). The patient has continued to experience intermittent anemia and gastrointestinal bleeding since this finding.

## Discussion

The described pathophysiology of Heyde’s syndrome has been evolving with a proposed mechanism of shearing forces on VWF multimers generating an acquired von Willebrand coagulopathy and subsequent bleeding causing moderate to severe anemia. Our case of Heyde’s syndrome involves severe subaortic stenosis and gastrointestinal AVMs identified by pill endoscopy. This patient did not have typical echocardiographic findings of hypertrophic cardiomyopathy or a family history of hypertrophic cardiomyopathy and was instead found to have a subaortic membrane. However, the pathophysiology is similar, with both involving left ventricular outflow tract obstruction. Furthermore, hypertrophic cardiomyopathy patients have been shown to have a direct relation of reduction in VWF multimers to the magnitude of outflow tract obstruction [10]. Subaortic membranes may mimic the left ventricular outflow tract obstruction of hypertrophic cardiomyopathy, but may be discerned utilizing Doppler that demonstrates a fixed obstruction rather than dynamic [11].

80% of patients with Heyde’s syndrome that received aortic valve replacement had no recurrence of bleeding after 15 years [12]. Similarly, this patient’s anemia initially resolved with surgical correction of the subaortic membrane. This further supports the proposed mechanism of an acquired von Willebrand coagulopathy by abnormal shear stress. Additionally, it has been shown that a resting peak gradient of 15 mmHg is sufficient to impair VWF [13]. Our patient had an initial peak gradient of 73 mmHg and a post-myectomy pressure gradient of 15 mmHg.

After a prolonged outpatient course, her hemoglobin downtrended, the transvalvular aortic gradient increased, and she developed mild-moderate aortic regurgitation. These factors likely continue to push her hemostatic profile towards coagulopathy. As previously mentioned, aortic regurgitation can induce von Willebrand coagulopathy, which was supported by the VWF assay obtained when she had recurrence of gastrointestinal bleeding. Normally, VWF:RCo and VWF:Ag are well-correlated. Ristocetin induces platelet agglutination in the presence of high molecular weight multimers. The ratio of RCo:Ag is normally 1.0. It has been described that a ratio <0.7 is used as a reference for the diagnosis of a type 2A von Willebrand coagulopathy [14]. This remains the gold standard in identifying a cardiovascular disease-associated acquired type 2A von Willebrand coagulopathy. Unfortunately, VWF assays were not obtained prior to surgical intervention to allow for comparison.

## Conclusions

This case identifies an additional cardiac lesion that induces an acquired type 2A von Willebrand coagulopathy that is unique to current literature. A subvalvular aortic membrane causing aortic stenosis should be scrutinized along with the classic severe aortic stenosis symptoms for possible coagulopathy and need for early intervention. If doppler studies demonstrate a fixed obstruction, further evaluation for underlying structural abnormalities, such as hypertrophic obstructive cardiomyopathy, should be considered. Lastly, our case demonstrates that recurrence of bleeding events after surgical correction of causal lesions should prompt re-evaluation, as the majority of patients do not experience bleeding events after surgical intervention.
